# Letter to the editor regarding “disparities in telemedicine during COVID‐19”

**DOI:** 10.1002/cam4.5036

**Published:** 2022-07-15

**Authors:** Brian De, Sean Maroongroge, Grace L. Smith

**Affiliations:** ^1^ Department of Radiation Oncology The University of Texas MD Anderson Cancer Center Houston Texas USA; ^2^ Department of Health Services Research The University of Texas MD Anderson Cancer Center Houston Texas USA

**Keywords:** access, digital divide, pandemic, policy, telemedicine

We read with interest the study by Qian et al.^1^ who reported on use of outpatient oncology telemedicine at their institution after the start of the COVID‐19 pandemic. The disparity in telemedicine use among Hispanic, Spanish‐speaking, low‐income, and Medicaid‐insured patients likely reflected underlying socioeconomic barriers faced by these groups to accessing technologic and healthcare resources as well as the need to overcome a socioeconomic digital divide in the United States. However, the authors also found that resource barriers did not explain all disparities identified in the study, given that Asian patients also had lower telemedicine use but still had high rates of smartphone ownership and home broadband access.

We believe that this important study therefore not only highlights needs to overcome structural socioeconomic and resource barriers to oncology telemedicine access, but also points to opportunities to advance more tailored, patient‐centered approaches to incorporating telemedicine in cancer care. Increasing evidence suggests that variation in trust—or conversely, mistrust—in patient–physician relationships and care mediated by telemedicine is complex and multidimensional, even within and across racial and ethnic groups. Other studies of Hispanic, African–American, or Asian patients have found concerns such as worry about telemedicine access in the face of lack of insurance, the physical absence of or poorer communication by physicians, the inability to monitor physician qualifications, and unreliability of privacy/confidentiality.^2^, ^3^ Preferences about mode of telemedicine delivery also vary in prior studies. One study showed that Asian patients were more likely than White patients to choose video visits but less likely to choose telephone visits.^4^ In other study settings, African American and Hispanic patients preferred audio‐only visits.^5^

Yet cancer patients express that telemedicine represents a convenient, valuable option.^2^, ^6^ Our recent study demonstrated that a majority of patients receiving radiation oncology care after the start of the COVID‐19 pandemic were managed through a hybrid telemedicine and in‐person management approach, and that utilization of telemedicine or in‐person management was almost equally influenced by patient‐level and physician‐level characteristics.^7^ Therefore, using shared decisions between patients and their physicians to implement hybrid approaches may represent an advance toward fluidly integrating in‐person and telemedicine care to optimize the quality of clinical management and simultaneously promote patient‐centered care.

Finally, it is important to underscore that the success of a tailored approach integrating video or audio telemedicine as a routine, acceptable, and patient‐centered component of care for vulnerable populations remains contingent on ongoing legal, payment, and privacy policy decisions about future availability of telemedicine services. Payment parity between in‐person, video, and audio‐only telemedicine visits is a temporary provision of the 1135 waivers created at the start of the COVID‐19 public health emergency (PHE). Public and private payers have begun reducing or eliminating reimbursement for audio‐only visits. Awareness is needed that such policy changes could impact the options and preferences influencing patients' and physicians' shared decisions about how to integrate telemedicine into patients' care. With an eye toward the post‐pandemic era, we laud the study by Qian et al. for highlighting the need for continued attention on how the actively changing landscape of oncology telemedicine could exacerbate disparities in cancer care or pose a critical opportunity to enact durable solutions to mediate these disparities at the patient‐provider, system, and policy levels.

## AUTHOR CONTRIBUTIONS

Conception or design of the work: De, Smith. Drafting the article: All authors. Critical revision of the article: All authors. Final approval of the version to be published: All authors.

## CONFLICT OF INTEREST

BD reports consulting honoraria from Sermo, Inc.

## Data Availability

N/A

## References

[1] Qian AS , Schiaffino MK , Nalawade V , et al. Disparities in telemedicine during COVID‐19. Cancer Med. 2022;11:1192‐1201. doi:10.1002/cam4.4518 34989148 PMC8855911

[2] Meno M , Abe J , Fukui J , Braun‐Inglis C , Pagano I , Acoba J . Telehealth amid the COVID‐19 pandemic: perception among Asian, native Hawaiian and Pacific islander cancer patients. Future Oncol. 2021;17(23):3077‐3085. doi:10.2217/fon-2021-0136 34102878 PMC8202507

[3] George SM , Hamilton A , Baker R . Pre‐experience perceptions about telemedicine among African Americans and Latinos in south Central Los Angeles. Telemed E‐Health. 2009;15(6):525‐530. doi:10.1089/tmj.2008.0152 PMC2956566 19566397

[4] Reed ME , Huang J , Graetz I , et al. Patient characteristics associated with choosing a telemedicine visit vs office visit with the same primary care clinicians. JAMA Netw Open. 2020;3(6):e205873. doi:10.1001/jamanetworkopen.2020.5873 32585018 PMC7301227

[5] Chen J , Li KY , Andino J , et al. Predictors of audio‐only versus video telehealth visits during the COVID‐19 pandemic. J Gen Intern Med. 2021;37:1138‐1144. doi:10.1007/s11606-021-07172-y 34791589 PMC8597874

[6] Nguyen M‐LT , Garcia F , Juarez J , et al. Satisfaction can co‐exist with hesitation: qualitative analysis of acceptability of telemedicine among multi‐lingual patients in a safety‐net healthcare system during the COVID‐19 pandemic. BMC Health Serv Res. 2022;22(1):195. doi:10.1186/s12913-022-07547-9 35164746 PMC8842908

[7] De B , Fu S , Chen YS , et al. Patient, physician, and policy factors underlying variation in use of telemedicine for radiation oncology cancer care. Cancer Med. 2022;11(10):2096‐2105. doi:10.1002/cam4.4555 35297210 PMC9119354

